# Drosophila Eggshell Production: Identification of New Genes and Coordination by Pxt

**DOI:** 10.1371/journal.pone.0019943

**Published:** 2011-05-26

**Authors:** Tina L. Tootle, Dianne Williams, Alexander Hubb, Rebecca Frederick, Allan Spradling

**Affiliations:** 1 Department of Anatomy and Cell Biology, Roy J. and Lucille Carver College of Medicine, University of Iowa, Iowa City, Iowa, United States of America; 2 Department of Embryology, Howard Hughes Medical Institute/Carnegie Institution, Baltimore, Maryland, United States of America; Instituto Nacional de Câncer, Brazil

## Abstract

Drosophila ovarian follicles complete development using a spatially and temporally controlled maturation process in which they resume meiosis and secrete a multi-layered, protective eggshell before undergoing arrest and/or ovulation. Microarray analysis revealed more than 150 genes that are expressed in a stage-specific manner during the last 24 hours of follicle development. These include all 30 previously known eggshell genes, as well as 19 new candidate chorion genes and 100 other genes likely to participate in maturation. Mutations in *pxt*, encoding a putative Drosophila cyclooxygenase, cause many transcripts to begin expression prematurely, and are associated with eggshell defects. Somatic activity of Pxt is required, as RNAi knockdown of *pxt* in the follicle cells recapitulates both the temporal expression and eggshell defects. One of the temporally regulated genes, *cyp18a1*, which encodes a cytochromome P450 protein mediating ecdysone turnover, is downregulated in *pxt* mutant follicles, and *cyp18a1* mutation itself alters eggshell gene expression. These studies further define the molecular program of Drosophila follicle maturation and support the idea that it is coordinated by lipid and steroid hormonal signals.

## Introduction

**Figure 1:**
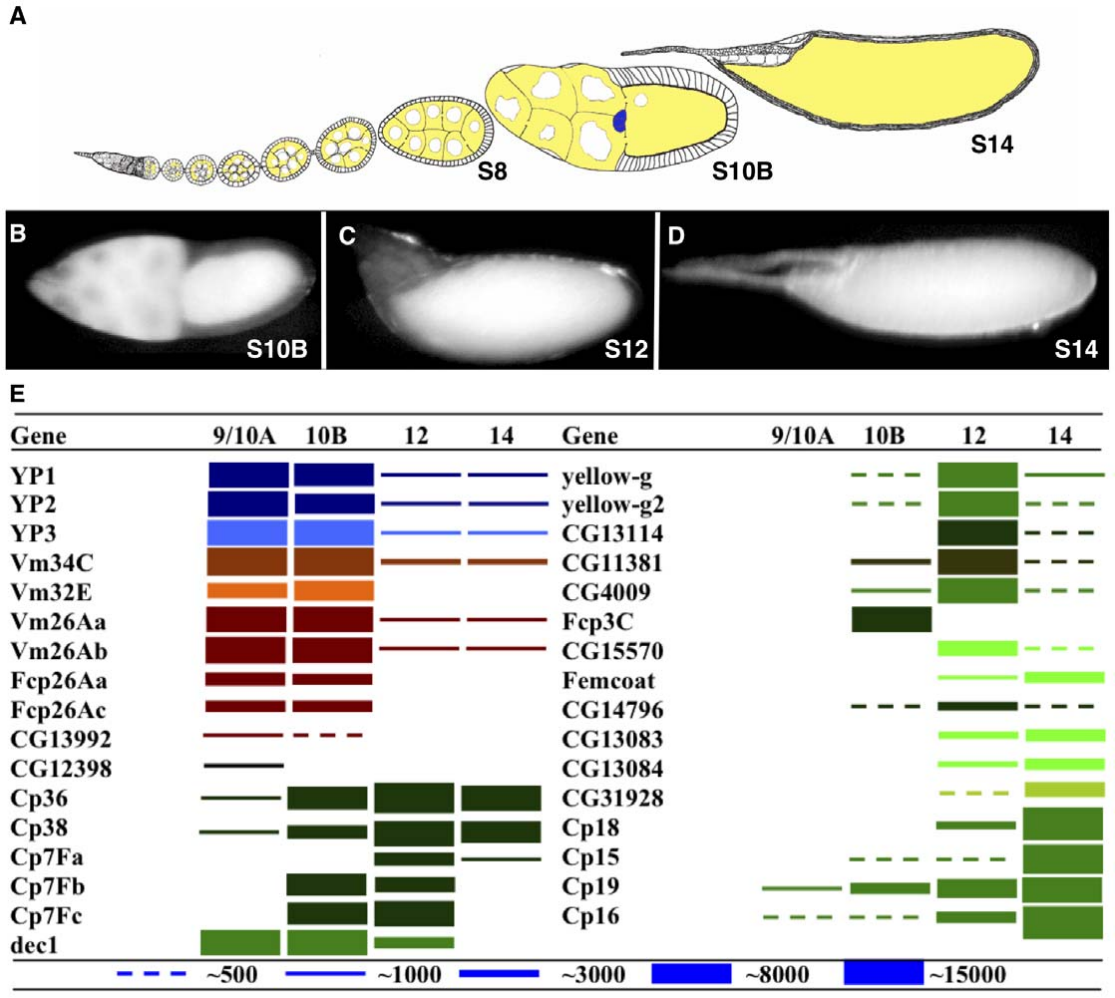
Temporally regulated expression of known eggshell protein transcripts. (A) An ovariole containing sequentially developing ovarian follicles ranging between stage 1 and stage 14 is depicted in cross section, revealing germline (yellow) cells comprising 15 nurse cells and an oocyte, surrounded after stage 7 by about 700 epithelial follicle cells. (B–D) Images of hand dissected stage 10B (B), stage 12 (C) or stage 14 (D) follicles, used for RNA isolation and microarray analyses. (E) The expression patterns determined from the microarray data of all 30 previously known eggshell (and 3 yolk) protein transcripts are depicted graphically. Adjacent genes with the same color are clustered in the genome. The full dynamic range of these changes is underestimated by the diagram, but can be found in Table S1. Accession numbers are listed in Table S1.

Drosophila ovarian follicles mature during their final day of development into a functional oocyte encased in a multi-layered shell capable of protecting and nurturing the developing embryo [1], [2], [3]. The oocyte, 15 germline-derived nurse cells and more than 700 somatic follicle cells work together during these stages, termed stages 8–14, to complete the egg (Figure 1A–D). Triggered by an internally generated steroid hormone signal, each follicle during stage 8 begins to take up yolk and to synthesize the first eggshell layer known as the vitelline membrane. Production of the chorion commences in stage 10B with follicle cell-specific amplification of several chorion structural gene clusters followed, during stages 11–14, by precisely programmed gene-specific transcriptional activation. Precise cell movements and intercellular signals during this period shape the main body of the eggshell, as well as its micropyle and dorsal respiratory appendages [4], [5]. In a process that is closely tied to eggshell production, the egg transmits anterior-posterior and dorsal-ventral patterning information from mother to embryo [6], [7]. For example, the Nudel serine protease is required both to cross-link the vitelline membrane [8], and to cleave Gastrulation Defective protein, an essential step in transmitting maternal dorsal-ventral patterning information [8]. Because of its cellular simplicity, sophisticated information content and exquisite patterning, the maturing follicle has become an attractive system for analyzing differentiation, gene regulation and morphogenesis [9], [10].

Eggshell proteins have been identified by electrophoresis (reviewed in [2]) and by mass spectroscopy [11]. Genes encoding approximately 30 such proteins are currently known, many located in clusters that are specifically amplified in follicle cells prior to expression (reviewed in [3]). One characteristic of the known eggshell genes is a very high degree of temporal and spatial regulation. Yolk and vitelline membrane proteins are synthesized during stages 9–10. “Early” chorion genes such as *Cp36* (FBgn0000359) and *Cp38* (FBgn0000360) are active only during stages 11–12, “middle” chorion genes such as *Cp19* (FBgn0000356) and *Cp16* (FBgn0000356) during stages 13–14, and “late” chorion genes such as *Cp18* (FBgn0000357) and *Cp15* (FBgn0000355) during only stage 14. Chorion genes are also spatially regulated within the follicle [12], [13], [14]. Several proteins including Dec-1 (FBgn0000427) and Cp36 move between eggshell layers as part of the morphogenetic process [15], [16], [17]. Steroid signals initiate maturation at stage 8 [18], and mediate several subsequent events including gene amplification and expression [19], [20]. Nonetheless, the regulation of eggshell gene expression remains poorly understood.

Prostaglandins (PGs), short-acting lipid hormones derived from arachidonic acid by the action of cyclooxygenase, play multiple roles in mammalian follicle development [21]. Some of these roles may have been conserved during evolution because pharmocologic inhibition of PG synthesis blocks follicle maturation in mice, zebrafish, silkmoth, and Drosophila [22], [23], [24], [25]. There are two enzymes responsible for generating PGs in higher animals, termed cyclooxygenase 1 and 2 (COX1 and COX2; aka prostaglandin H-synthase 1 and 2; PGHS1 and PGHS2). Both genetic knockdown and pharmacologic experiments implicate COX2 in mediating mouse follicle development [26], [27]; while COX1-like enzymes appear to be involved in the lower animals [23], [24], [25]. The *pxt* gene (FBgn0261987) has been proposed to encode a Drosophila cyclooxygenase [25]. Exogenous PGE_2_ and PGF_2alpha_ can overcome the effects of the inhibitors in mice [22], [27], while PGF_2alpha_ rescues development in zebrafish, silkmoth, and Drosophila [23], [24], [25]. While it is clear that PGs influence follicle development across diverse animals, the mechanisms of PG action in invertebrates remain largely unknown.

Here we have used characteristic temporal regulation to identify new eggshell genes and to identify genes involved in multiple aspects of egg maturation. One of these, Cyp18a1 (FBgn0010383), was recently shown to encode a cytochrome P450 protein and to mediate the breakdown of the steroid hormone ecdysone [28]. Both *pxt* mutation and *cyp18a1* misexpression alter the timing of chorion gene expression and disrupt eggshell morphogenesis. Our studies support the view that a Pxt-dependent signal acts along with ecdysone to coordinate eggshell production.

## Materials and Methods

### Fly strains

Fly stocks were maintained at 20–25°C on standard cornmeal-agar-yeast food. pxt[f01000], pxt[EY03052] and UASp Pxt strains were described previously [25]. The RNAi stock for knocking down *pxt* expression was obtained from the Vienna Drosophila RNAi Center (transformant identification number 14379). The RNAi line was crossed with c355 and t80 GAL4 driver lines (Bloomington Drosophila Stock Center) to assess the roles of Pxt in the soma during follicle development. RNAi crosses and progeny were maintained at 27.5°C. The *Cyp18a1* allele utilized was d01488 (Exelixis Collection at the Harvard Medical School).

### RNA isolation and microarray analysis

Specific staged follicles were hand dissected in room temperature Grace's medium (Lonza). The media was removed, and the tissue was ground with a plastic pestle (Kontes) in 100 µl of Trizol (Invitrogen), quick frozen in liquid nitrogen, and stored at −80°C until enough tissue was isolated. RNA was extracted according to the manufacturer's instructions. The Johns Hopkins Microarray Core Facility performed the microarray analyses using 10 µg of RNA to probe *Drosophila* version 2.0 Affymetrix chips. Microarrays were performed at least twice using independently prepared RNA samples for each stage and genotype. Microarrays were normalized to the same total expression, which is believe to change little during egg maturation due to the abundant RNA content of the germ cells. Correction factors were quite small (less than 50%) and were negligible except for highly expressed genes (top 10%). For a gene to be scored as temporally regulated, its transcript level had vary more than 4-fold during maturation (stages 9–10A to S14), and to show peak expression above 150. However, most of the selected genes far exceeded these parameters. All data is MIAME compliant and the raw data has been deposited in GEO.

A subset of the microarray results was verified by semi-quantitative RT-PCR. Stage-specific follicle RNA was isolated, as described above. cDNA was synthesized using Reverse Transcription Kit (Promega), and 0.25–3 µL of cDNA was used per PCR reaction with primers for specific genes. Control reactions were performed at identical cDNA concentrations and PCR conditions. Primer sequences are available upon request. PCR reactions were analyzed using agarose gel electrophoresis and imaged with a UVP BioDocIt system.

### Eggshell analyses

For all embryo collections, flies were allowed to lay eggs on wet yeasted molasses plates (9% molasses, 2.2% agar, 1.8% of 5% tegosept solution) or grape juice plates (50% grape juice, 2% agar, 2% of 5% tegosept solution) for 30 minutes, the plate was replaced with a freshly yeasted plate and flies were allowed to lay eggs overnight. To quantify chorion and dorsal appendage defects, the laid eggs were examined on the plates under a dissecting scope. To assess for vitelline membrane defects a neutral red dye uptake assay was performed essentially as described [8].

### Immunofluorescence

Whole mount samples were fixed with 4% paraformaldehyde for 10 minutes and processed using standard procedures [29]. Goat anti-Nudel DL-20 or DC-16 (Santa Cruz) were used 1∶50. Donkey anti-goat conjugated to Alexa 488 or Alexa 568 (Molecular Probes) was used at 1∶1000. To visualize DNA, DAPI (1 µg/ml) was added to the final wash before mounting the samples in mounting media (5 mg/ml phenylenediamine in 1×PBS and 50% glycerol).

### Microscopy

Dissected stages of follicle development and laid eggs were imaged in Grace's media (Lonza) on an Olypmus SZ61 dissecting scope with a DP25 color camera and DP2-BSW software.

Immunofluorescent samples were imaged with a 20× (NA 0.7) lens on a CARV- spinning disc confocal with a Hamamatsu EM-CCD Digital ImagEM Camera (C9100-13). All confocal images are projected *z*-stacks produced using ImageJ and cropped/rotated in Photoshop.

## Results

### Identification of temporally regulated genes during egg maturation

Since known eggshell genes are each expressed during characteristic temporal intervals of follicle development, we searched for additional temporally regulated follicle genes. RNA was prepared from groups of 100–300 hand-isolated ovarian follicles at 4 stages that span the period of eggshell production: S9-10A, S10B, S12, and S14 (Figure 1B–D). Each RNA population was analyzed using Affymettrix 2.0 Drosophila microarrays and genes whose transcripts were significantly modulated were identified (Methods).

**Figure 2:**
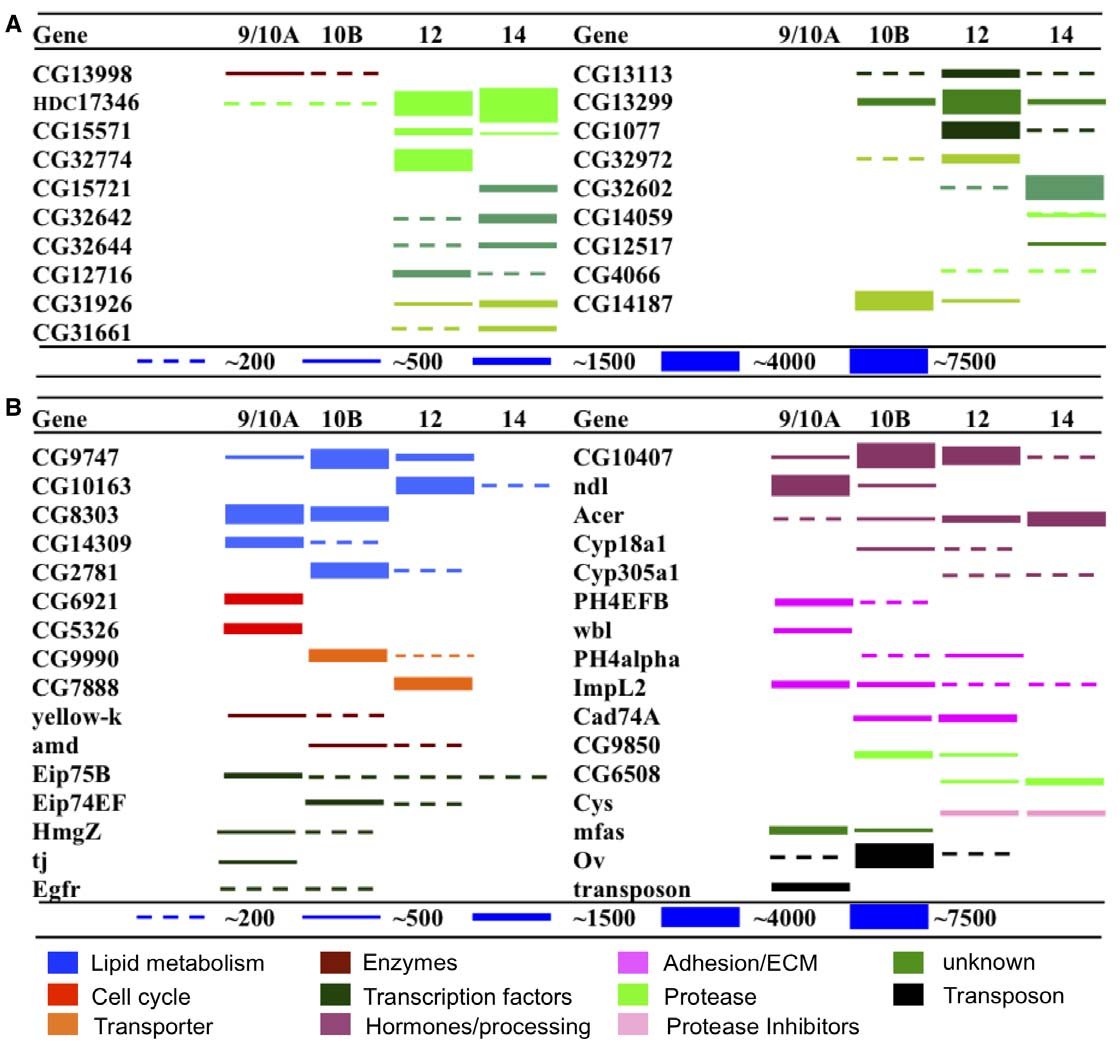
Expression of new putative eggshell protein and late follicle transcripts. (A) The temporal expression patterns of 19 new putative eggshell protein transcripts as determined by microarray analyses. The full dynamic range cannot be depicted and the scale differs from Figure 1. Numerical values can be found in Table S2. Adjacent genes with the same color are clustered in the genome. (B) The temporal expression patterns of 32 genes in various categories from among the differentially expressed genes identified (for the full list see Table S3). Transposon = Tirant (1640955_s_at). Accession numbers are listed in Tables S2, S3.

The results observed with the 30 genes encoding known eggshell proteins suggested that this approach was both accurate and highly sensitive (Figure 1E). Transcripts from all eggshell genes were detected and their stage-specificities of expression were accurately recapitulated (Table S1), validating the reliability of our experimental paradigm. In addition, 120 additional genes were found whose transcripts also accumulated in a stage-specific pattern (Figure 2, Tables S2, S3). We verified the expression behavior of a subset of these new genes using RT-PCR (Figure S1).

### Identification of genes encoding new candidate eggshell proteins

Known eggshell protein genes display several distinctive characteristics in addition to stage-specific expression. In many cases they are present in gene clusters, are highly expressed but only in the ovary, and encode proteins that undergo relatively rapid evolutionary change. Based on these characteristics, we identified 19 of the new stage-specific genes as likely to encode eggshell proteins (Figure 2A, Figure S1, Table S2).

Eleven of these genes reside within clusters, like most previously characterized chorion genes (Figure 2A, Table S2). For example, within the 26A vitelline membrane gene cluster we identified a sixth gene, *CG13998* (FBgn0040949), which is expressed in a pattern consistent with a role in vitelline membrane formation. At the 4B4 gene cluster, near the eggshell genes *femcoat* (FBgn0041252) and *CG15570* (FBgn0029697) [11], we identified three new putative eggshell genes, *CG32774* (also known as *muc4B*, encoding a mucin-like protein; FBgn0052774), *CG15571* (FBgn0029696), and *HDC17346* (FBsf0000016774), a highly expressed RNA with an uncertain gene model. Thus, at least 5 chorion proteins appear to be encoded at this locus. Additionally, in region 22A2, adjacent to the known chorion gene *CG31928* (FBgn0051928) [11], we identified two new putative eggshell genes *CG31926* (FBgn0051926) and *CG31661* (FBgn0051661). All three of these proteins are predicted to encode aspartic proteases. Within the 30B minor amplified domain [30], which is located adjacent to the chorion gene *CG13114* (FBgn0032127) [11], we found that *CG13113* (FBgn0032126) undergoes stage-specific expression, but at a quantitatively lower level. Finally, the genes *CG15721* (FBgn0030438), *CG32642* (FBgn0052642), *CG32644* (FBgn0052644) and *CG12716* (FBgn0030439) constitute an entirely new cluster of putative eggshell genes in region 11D5. As with the other chorion clusters, the genes are expressed during similar but not identical intervals. *CG32644* encodes another mucin-related protein (Mur11D), while CG15721 and CG12716 contain protease inhibitor domains, including a follistatin-like domain.

Although not clustered, the remaining new candidates displayed other evidence consistent with a role in the eggshell. For example, *CG32602* (*mucin12Ea*; FBgn0052602) [31] is abundantly expressed at stage 14 and encodes another mucin-like protein consisting largely of a 13-amino acid repeating motif (Figure 2A, Table S2). A chorion gene has long been suspected to reside in this location, since follicle cell mRNA hybridizes *in situ* to 12E as strongly as to the major chorion cluster at 7F [32]. One of the remaining candidate eggshell genes, *CG1077* (FBgn0037405) is predicted to possess anti-microbial activity. Another, CG32972 (FBgn0028905) has a fasciclin-like domain and a region with homology to yolk granule proteins. All the candidate chorion proteins were expressed specifically in the ovary (Figure S1; see also [33]).

### Identification of additional differentially expressed genes

The microarray analyses identified about 100 other genes whose steady state RNA levels changed more than fourfold during egg maturation (Figures 2B, Table S3). Some encode proteins known, through earlier genetic and molecular studies, to participate in follicle development. These temporally regulated genes encode proteins that are predicted to function in several broad classes, including lipid metabolism, membrane transport, cell cycle regulation, intercellular communication, enzymatic activity, adhesion and transcriptional regulation (Table S3). We also identified new candidate genes that may play important roles during follicle development based on their time of expression (Table S3), tissue-specificity (Figure S1, see also [33]) and predicted function. For example, metabolic genes may help provision the egg with lipid and glycogen yolk. Cyp18a1, one of the predicted enzymes, has been implicated in ecdysone degradation [28], and transcription factors such as Eip75B (FBgn0000568) and Eip74EF (FBgn0000567) may mediate steroid responses [18]. Additionally, we discovered many new genes that are expressed only during stage 14 (Table S2, S3); these may include genes that function to prepare the egg for ovulation, and/or activation during passage in the oviduct.

### Pxt is required for correct temporal gene expression

Mutations in genes that regulate egg maturation might alter the precise temporal expression program described above. Consequently, we examined gene expression in stage 10B-14 follicles isolated from *pxt* mutant females using the same protocol as for wild type. Two different *pxt* alleles, f01000 and EY03052, were studied, both of which reduce *pxt* RNA to below detectable levels in mutant ovaries [25].

**Figure 3:**
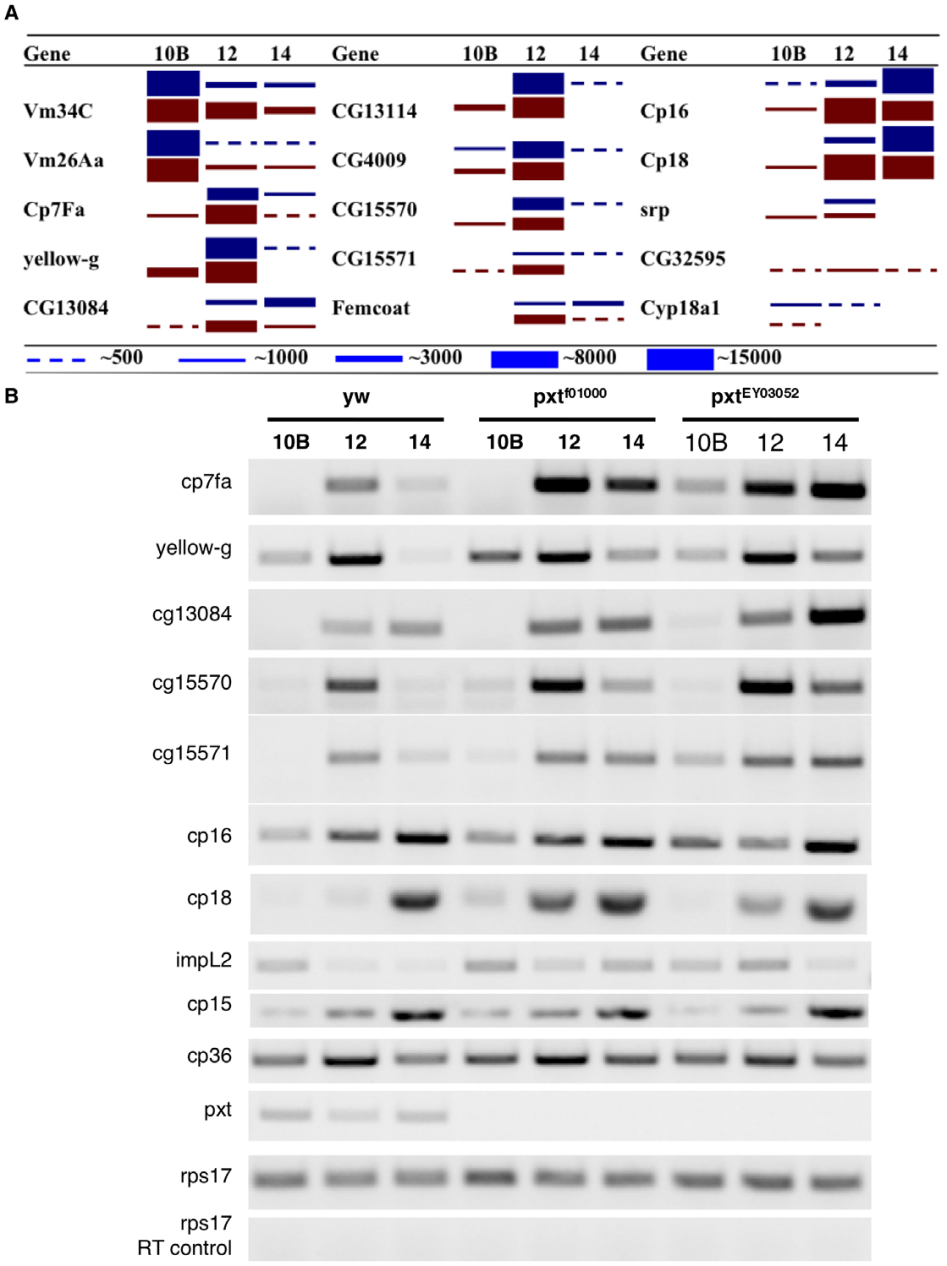
*pxt* mutants disrupt the timing of gene expression during follicle development. (A) Diagram similar to those in Figures 1–2 (but scales differ) summarizes microarray data on the stage-specific expression of 15 eggshell genes in wild type (blue) and *pxt^f01000^* (maroon) mutant follicles. At least one gene is over-expressed (*Cp7Fa*), while transcripts from others begin to accumulate prematurely (*CG13114*, *cp16*), and over a broader temporal range (*imp-L2*). (B) RT-PCR experiments confirming the changes observed in the microarrays for a subset of genes.

Loss of Pxt substantially changed the temporal program of gene expression during late oogenesis. Transcripts from one of the early chorion genes, *Cp7Fa* (FBgn0014464), are elevated 2–3 fold, an effect confirmed by RT-PCR (Figure 3A–B). Several genes involved in vitellogenesis, such as *vm34C* (FBgn0003983), *vm26Aa* (FBgn0003979), and *impL2* (FBgn0001257), shut off RNA levels more slowly than in wild type (Figure 3A–B). Transcripts from other chorion genes show a broader period of expression, appearing earlier and/or persisting longer. For example, *yellow-g* (FBgn0041709) is detected prematurely in stage10B *pxt^f01000^* follicles, and persists into stage 14. *CG13114*, *CG4009* (FBgn0038469), and *cp18* appear prematurely in stage10B, while the onset of *CG13084* (FBgn0032788), *cp16*, *femcoat*, *CG15570* and *CG15571* expression is also shifted earlier and/or prolonged. The expression of other genes, including many non-chorion genes was also altered in timing and amount in *pxt* mutant follicles (Figure 3, Figure S2). Pxt mutation severely reduced the expression of a few genes whose transcripts are normally constant during stage 10B-14 follicles, including *gstD1* (reduced 20-fold; see also Figure S2; FBgn0001149), *CG12273* (reduced 25-fold; FBgn0016762) and *rala* (reduced 40-fold; FBgn0015286). Thus, loss of Pxt alters the temporal program of gene expression associated with egg maturation.

### *Pxt* follicles synthesize a defective eggshell

**Figure 4:**
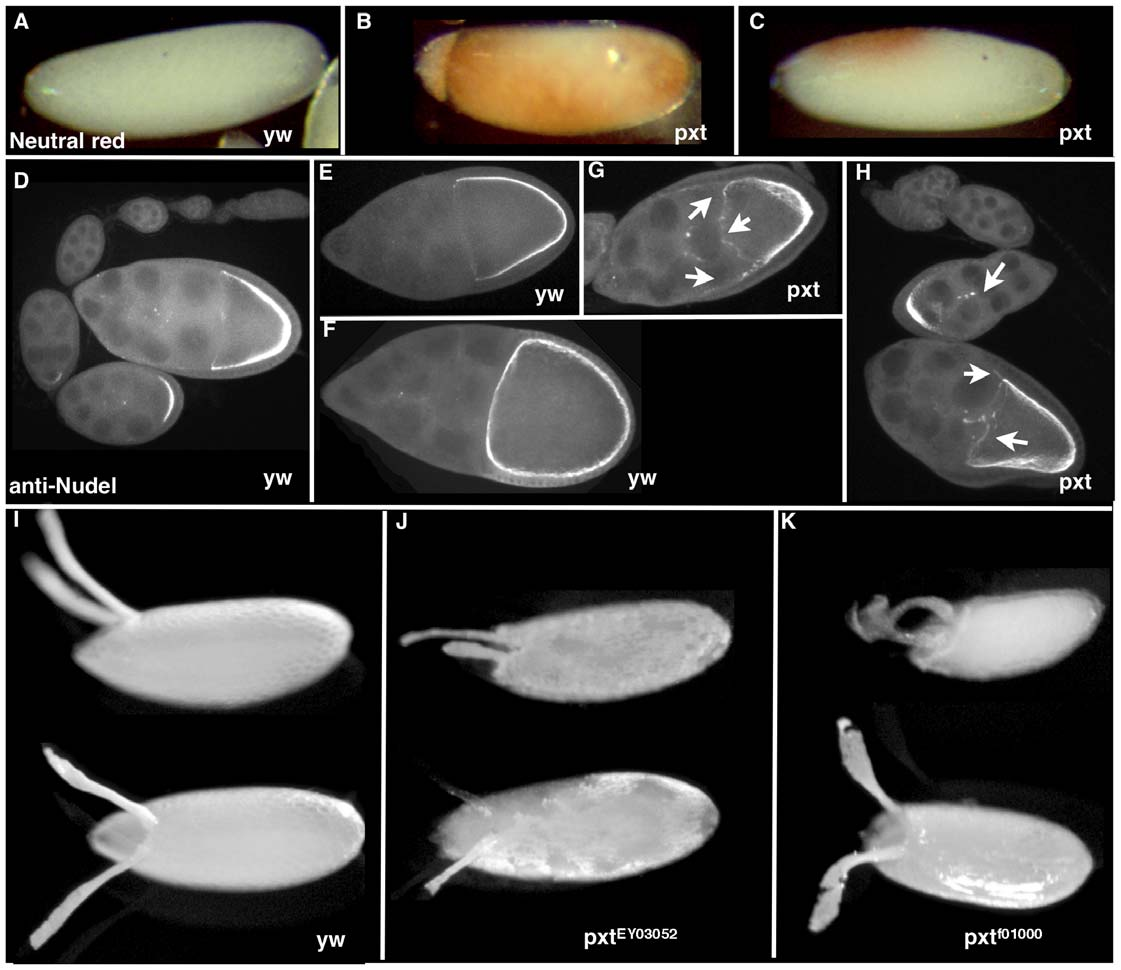
*pxt* mutants disrupt eggshell production. (A–C) Light microscopic images of laid, dechorionated eggs incubated with neutral red dye. Wild-type eggs (*y w*) are impermeable and exclude the dye (A), while *pxt* mutant eggs often appear abnormal, especially at the anterior end, and have permeability defects allowing dye uptake (B–C). (D–H) Nudel expression. In wild type (*y w*) main body follicle cells secrete Nudel around the oocyte during stages 8–10A (D–E) and centripetally migrating follicle cells secrete it at the anterior of the oocyte during stage 10B. In contrast, in *pxt* mutant follicles, Nudel is secreted prematurely, both at the anterior and away from the oocyte (F–G, arrows). (I–K) Light microscope images of laid eggs. The outer shell layers (chorion) are morphologically abnormal in laid *pxt* mutant eggs. Dorsal appendages are short, and misformed (J), and the main body chorion is uneven and rough (J–K), compared to wild-type laid eggs (I).

We examined the structure of *pxt* mutant eggs to determine if the expression changes observed above were associated with eggshell defects. To assess the vitelline membrane integrity, we examined whether dechorionated eggs are impermeable to neutral red dye. 41% (n = 179) of laid *pxt^f01000^* eggs and 34% (n = 240) of laid *pxt^EY03052^* eggs took up dye, indicating a vitelline membrane defect, compared to only 1.7% (n = 675) of *yw* eggs (Figure 4B–C, compared to A). While dye uptake was frequently localized near the anterior egg region, 25–30% of *pxt* mutant eggs took up dye along the entire anterior-posterior axis. Thus, examination of eggs laid by *pxt* mutants revealed defects in eggshell structure, supporting the idea that Pxt-mediated temporal gene regulation is necessary for proper eggshell formation.

Since Nudel (FBgn0002926) protein is known to be involved in vitelline membrane cross-linking [8], we examined its expression in wild type and *pxt* mutant follicles using specific antisera. Nudel is expressed and secreted by the follicle cells adjacent to oocyte starting at S8 (Figure 4D). As more follicle cells contact the oocyte the number of follicle cells secreting Nudel increases (S9-10A; Figure 4E); at S10B, when the centripetal follicle cells migrate over the anterior of the oocyte, Nudel expression encases the oocyte (Figure 4F). Consistent with the possibility that Pxt acts upstream in this pathway, in *pxt* mutants, follicle cells that have not yet reached the oocyte secrete Nudel (Figure 4G–H, arrowheads). The protein prematurely accumulates at the oocyte anterior in S9 follicles, and aggregates of Nudel protein remain between the nurse cells. Thus, Pxt is required for the proper temporal and spatial regulation of Nudel accumulation.

Pxt also was found to be necessary for normal chorion production. The morphology of the chorion and dorsal appendages was altered in all *pxt* eggs examined (N = 164; Figure 4J–K, compared to I). The most common defects were shortened and unequal dorsal appendages, residual nurse cell material due to incomplete dumping, and uneven chorion deposition resulting in a rough appearance. Thus, the altered pattern of gene expression detected by microarray correlates with defects in both the vitelline membrane and chorion layers of laid *pxt* mutant eggs.

### Pxt is required in the follicle cells to regulate eggshell production

**Figure 5:**
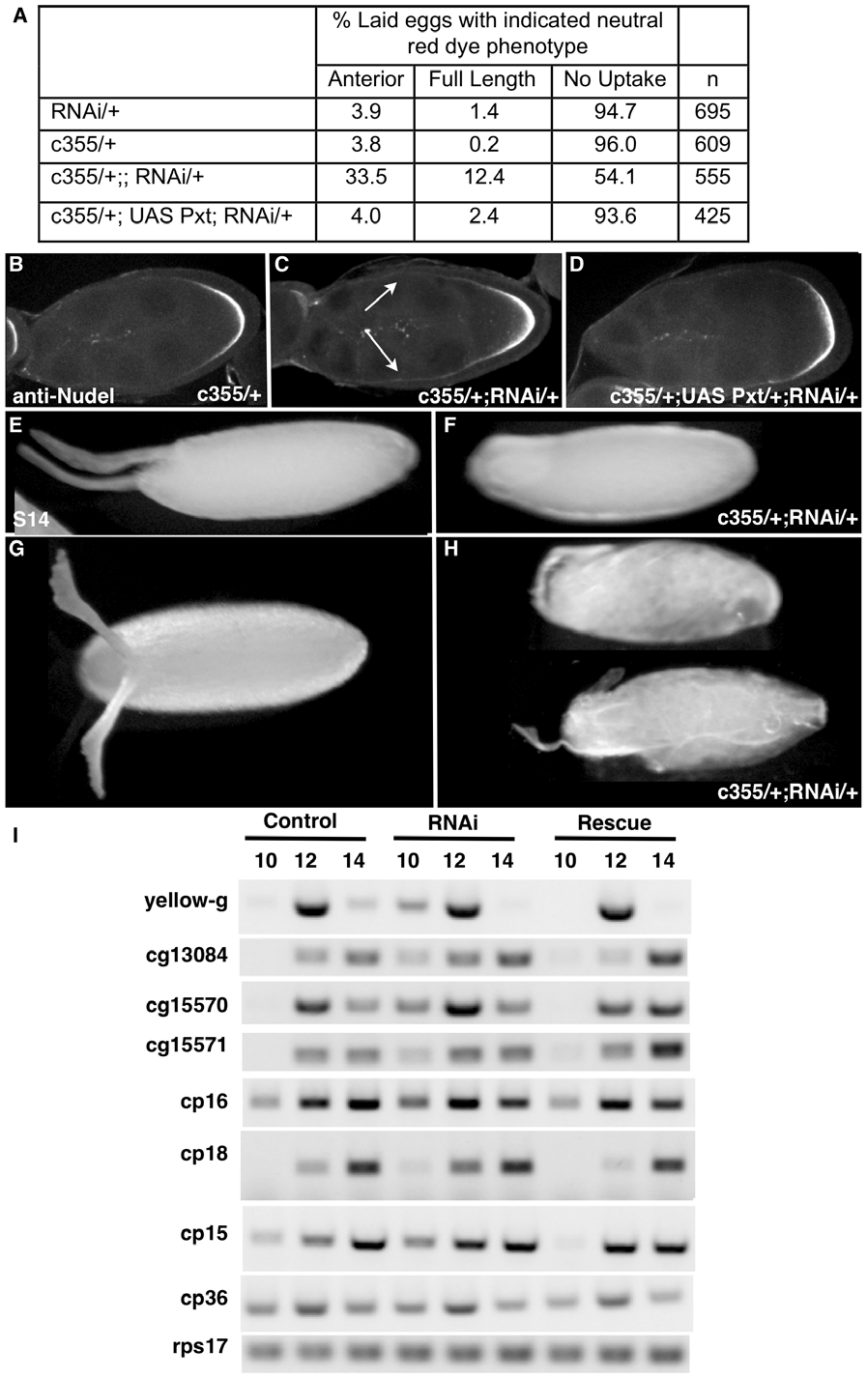
Pxt is required in the follicle cells for eggshell production. (A) Table reporting the number of laid, dechorionated eggs that take up neutral red dye. Control eggs, with the GAL4 driver or the UAS Pxt RNAi construct alone, are impermeable and exclude the dye, while eggs with somatic knockdown of Pxt expression by RNAi (c355/+; RNAi/+) have permeability defects allowing dye uptake. These defects can be rescued by co-expression of the full-length *pxt* cDNA (c355/+; UAS Pxt/+; RNAi/+). (B–D) Nudel expression. Control stage 9 follicles express Nudel only along the main body follicle cells (B), while stage 9 follicles where Pxt RNAi is driven in the somatic cells exhibit Nudel expression away from the oocyte (C). Co-expression of Pxt restores normal Nudel expression (D). (E–F) Light microscope images of stage 14 follicles (E–F) and laid eggs (G–H). Control stage 14 follicles (E) and laid eggs (F) exhibit normal morphology and eggshell appearance. Dorsal appendages are missing, short and rounded, or misshapen when Pxt RNAi is driven in the somatic cells (F, H); additionally the outer chorion appears abnormal in laid eggs (H). (I) RT-PCR of staged follicles. RNAi knockdown of *pxt* recapitulates the temporal defects in eggshell gene expression seen in *pxt* mutants (see Figure 3). Co-expression of Pxt with the RNAi construct restores the normal timing of gene expression.

*pxt* is expressed in both the germline and the somatic cells during oogenesis [25]. To investigate which cells require *pxt* function for normal eggshell production we knocked down *pxt* expression specifically in follicle cells. Knock down of *pxt*, using the ubiquitous somatic GAL4 lines t80 or c355 to drive UAS-*pxt* RNAi expression at 27.5°, caused eggshell defects similar to those observed in *pxt* mutants. For example, 45.9% of the eggs laid by *pxt* RNAi-expressing females take up neutral red dye (Figure 5A). As with *pxt* mutants, some permeability defects were restricted to the egg anterior while others extended along the full-length of the egg. Co-expression of UAS-*pxt* along with the RNAi construct reduced the frequency of dye-uptake to control levels (Figure 5A). Like *pxt* mutations, somatic knockdown of *pxt* also caused premature Nudel expression/secretion during stage 9 by follicle cells that had not yet reached the oocyte (Figure 5C compared to B). Nudel expression was normalized by expressing UAS-*pxt* along with *pxt*-RNAi (Figure 5D). Finally, stage 14 *pxt* RNAi follicles and laid eggs exhibited missing or abnormal dorsal appendages and defective chorions (Figure 5F,H compared to E, G).

Knocking down *pxt* expression using somatic GAL4 drivers reduced fertility, and most females became sterile within 4 days. Fertility was not compromised if a full-length *pxt* cDNA was co-expressed with *pxt* RNAi (data not shown). In contrast to *pxt* mutants, however, defects in nurse cell dumping and egg elongation were not observed in follicles from females expressing *pxt* RNAi in the follicle cells. Thus the eggshell defects observed in *pxt* mutants are not simply a consequence of a block in nurse cell dumping or decreased egg elongation.

We carried out RT-PCR experiments using follicular RNA from control and RNAi-expressing flies to determine if somatic disruption of *pxt* expression causes similar changes in temporal gene expression to those observed in *pxt* mutant follicles (Figure 3). Many such changes were observed (Figure 5I). For example, the expression of *yellow-g*, *CG13084*, *CG15570*, *CG15571* and several other chorion genes began earlier in follicles from somatic *pxt* RNAi-expressing females, but these defects were rescued by co-expression of UAS-*pxt* (Figure 5I). We conclude that *pxt* is required in the follicle cells to coordinate the timing of gene expression during follicle maturation and for normal eggshell production.

### Cyp18a1 is required for normal chorion gene expression and eggshell production

**Figure 6:**
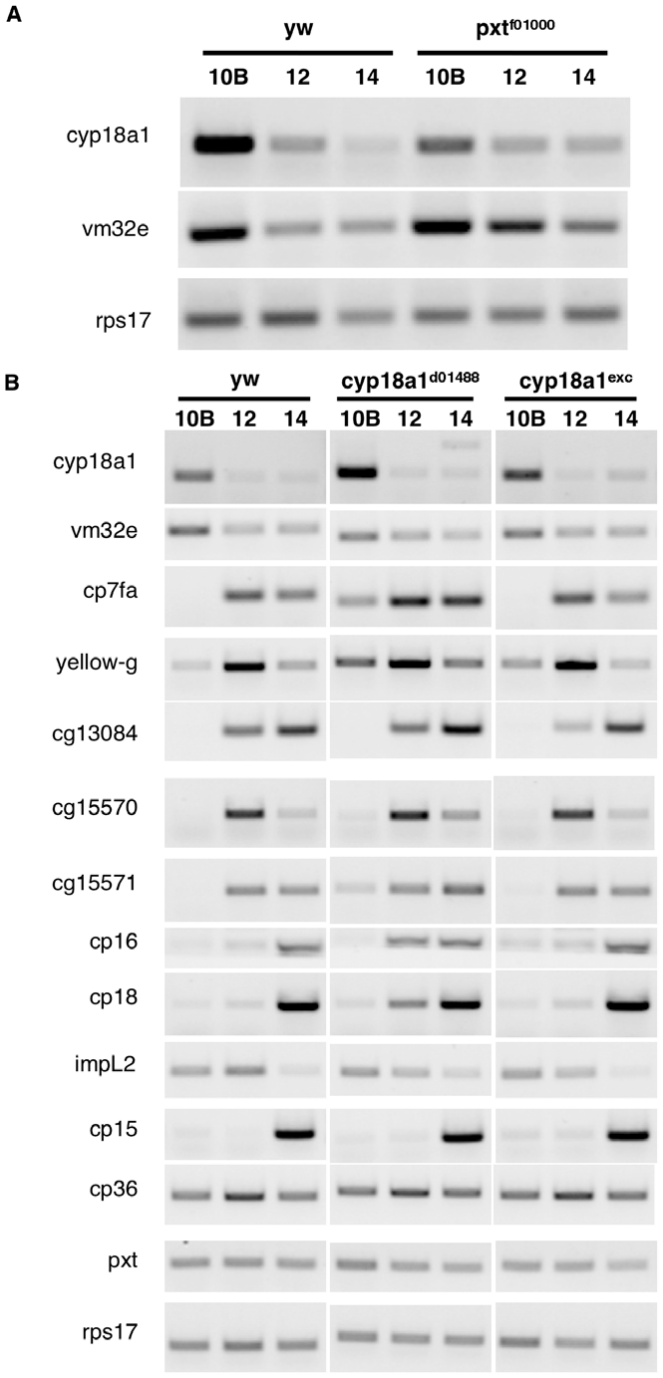
*Cyp18a1* is regulated by *pxt* and is required for eggshell production. (A–B) RT-PCR of staged follicles. In control follicles (*y w*) both *cyp18a1* and *vm32e* are expressed strongly at S10B and downregulated in later stages of follicle development (S12, S14) (A). In contrast, in *pxt* mutant follicles, *cyp18a1* expression is reduced at S10B compared to controls; conversely, *vm32e* expression is upregulated at S10B and fails to be properly downregulated in later stages (S12, S14) (A). The *d01488* insertion upstream of *cyp18a1* results in an approximately 2-fold increase in *cyp18a1* expression at S10B and the production of a novel isoform at S14. This misexpression of *cyp18a1* results in a mild decrease in *vm32e* expression, and *pxt-*like changes in eggshell gene expression (B). A precise excision of the insertion restores normal gene expression.

The cytochrome P450 protein encoded by *cyp18a1* catalyzes the 26-hydroxylation of ecdysteroids and their subsequent degradation [28]. Normally, *cyp18a1* transcripts increase during S10B and are subsequently down-regulated (Figure 6A). Both microarray and RT-PCR analyses reveal that mutations in *pxt* decrease *cyp18a1* transcript levels, especially at S10B (Figure 6A, Table S3). Thus, *pxt* mutation may cause ecdysone levels to rise prematurely during stage 10B by promoting a reduced level of ecdysone turnover.

The behavior of a known ecdysone target gene, *vm32e* (FBgn0014076) [34], is consistent with the idea that ecdysone levels are elevated in stage 10B *pxt* mutant follicles. The *vm32e* gene encodes a vitelline membrane protein that is expressed during stage 10. In *pxt* mutants, *vm32e* is more highly expressed during S10B and transcripts decline in abundance more slowly during stages 12–14 than is seen in wild-type follicles (Figure 6A).

The role of Cyp18a1 in eggshell production was further examined by identifying a mutation that perturbs its expression. The *P*-element insertion in strain *d01488*, which is located 186 bp upstream from the predicted promoter, increases *cyp18a1* RNA levels at S10B (Figure 6B). In addition, a novel transcript isoform is generated but only at S14 (Figure 6B). A precise excision of this element, *cyp18a1^exc^* restores normal *cyp18a1* expression. As expected if *cyp18a1* negatively regulates ecdysone target gene expression, *vm32e* expression is mildly reduced in *cyp18a1^d01488^* S10B follicles compared to wild type or *cyp18a1^exc^* follicles (Figure 6B).

We examined the effects of *cyp18a1^d01488^* mutation on several other genes involved in follicle maturation by carrying out RT-PCR using RNA isolated from *cyp18a1^d01488^* and *cyp18a1^exc^* follicles. The expression of several eggshell genes that were affected in *pxt* mutants and *pxt* RNAi were similarly altered in the *cyp18a1* mutant (Figure 6B compared to 3B and 5I). These data argue that the regulation of ecdysone stability is important for the temporal regulation of eggshell gene expression.

## Discussion

### Temporal regulation as criterion for gene identification

Our studies show that a large fraction of the genes involved in eggshell production can be identified by simply scoring for stage-specific changes in transcript levels during late oogenesis. Not only were virtually all of the previously known structural genes involved in producing yolk, the vitelline membrane and the chorion identified, but we also discovered at least 19 new candidate eggshell proteins (Tables S1, S2). Despite the simplicity of the protocol, the expression profiles revealed by our experiments agreed closely with previous studies, including the temporal programs reported by Fakhouri *et al.* (2006) for 10 minor chorion proteins as determined by whole mount *in situ* hybridization (Table S1). Many of the genes we identified as eggshell genes are included among 81 genes reported as candidate targets of Egfr signaling by Yakoby *et al.* (2008). Additionally, the expression of many genes involved in follicle maturation are spatially regulated within the folliclar epithelium [10], [11], [12], and our method could serve as an efficient pre-screen before undertaking such studies.

It is interesting to compare our results with studies of eggshell proteins isolated from whole ovaries and analyzed by mass spectrometry [11]. Our analyses confirm many of the 22 genes identified in this study, including *CG11381* (FBgn0029568), *CG13083* (FBgn0032789), *CG13084*, *CG13114*, *CG14796* (FBgn0025390), *CG15570*, *CG4009*, and *CG31928*. *CG3074* (FBgn0034709) and *CG13992* (FBgn0031756) are expressed at lower levels during stage 10 and may encode vitelline membrane proteins. The value of using temporal regulation as a criterion to identify eggshell components is highlighted by the fact that transcripts corresponding to the minor basement membrane components identified by Fakhouri *et al.* (2006) remain constant during eggshell formation (Table S3), suggesting that they derive from matrix material adherent to the purified eggshells, rather than representing true eggshell constituents.

### New eggshell components

Like the known eggshell genes, many of the 19 new candidate eggshell genes reside within existing or new gene clusters. For example, within the vitelline membrane gene cluster at 26A, we predict that *CG13998* encodes a novel Vm protein. CG13998 is expressed with the same stage specificity as other Vm proteins encoded within the 26A cluster, but is much less abundant. It may encode a rare or spatially restricted component of the vitelline membrane.

A number of the putative eggshell genes encode for proteins with mucin-like domains (CG32774/Muc4B, CG32642/Mur11D, CG32602/Muc12Ea). All three were scored as ovary-specific in FlyAtlas [33]. Mucin domains are heavily glycosylated and proteins with such domains often create a gel-like secretion. Mucins are known to function as components of chicken eggshells [35] and coat the reproductive tract in some animals [36]. CG32774/Muc4B is expressed mainly at S12. Based on the functions of mucin proteins and the timing of gene expression, we postulate the Muc4B may be a component of the wax layer that is located between the vitelline membrane and the chorion. The other mucin-like domain proteins are expressed at S14 and may mediate chorion hardening, protection against infection, or serve as a coating necessary for passage through the oviduct.

Another class of predicted eggshell genes encode for proteases and protease inhibitors. Such proteins have recently been shown to be a major class of chicken eggshell components [37]. CG31928, CG31926, and CG31661 are aspartic proteases. Proteases may act to process other chorion proteins into their mature form, and/or contribute to eggshell hardening. Previously, the protease CG31928 has been shown by *in situ* hybridization to exhibit a posterior restriction [11]. This raises the idea that proteases may be spatially restricted to alter chorion structure at specific regions that are important for subsequent function. It seems likely that other proteases may be restricted anteriorly, to the operculum area, to alter the eggshell structure to subsequently mediate larval hatching. In addition to proteases, we find that a number of protease inhibitors are eggshell candidates, including CG15721 (S14), CG12716 (S14), CG1077 (S12), and CG15418 (S12; FBgn0031554). Stage 12-expressed protease inhibitors may regulate eggshell proteases, while stage 14-expressed inhibitors may perform anti-microbial activities. Both the proteases and their inhibitors may also contribute to the embryo's ability to reutilize eggshell components for its development [37].

The nature of the peroxidase that crosslinks the eggshell [38] has been controversial. Various proteins have been suggested to function as the crosslinking peroxidase, including Pxd (FBgn0004577) [39] and Pxt [40]. We find *pxt*, the COX-like enzyme, is expressed and *pxd* is absent throughout all stages of egg maturation. In contrast, the eggshell protein and putative peroxidase CG4009 is very highly expressed during S12 (Table S1). We propose that CG4009 is the peroxidase that crosslinks the eggshell.

### Other temporally regulated genes during late follicle development

In addition to known and novel eggshell components, we identified many temporally regulated genes expressed during late follicle development. A number of putative lipid-processing genes exhibit stage specific expression, suggesting a role in yolk or pheromone production. *CG9747* (FBgn0039754) encodes for an acyl-CoA Δ11-desaturase, which is likely to desaturate palmitate; such enzymes are important for pheromone biosynthesis in other insects [41]. *CG8303* (FBgn0034143) is highly expressed as stages 9/10A and 10B, and encodes for an acyl-CoA reductase, which activates fatty acids by adding CoA. Additionally two Elvol (elongation of very long chaing fatty acids) encoding genes, *bond* (*CG6921*; FBgn0260942) and *CG2781* (FBgn0260942), are expressed at S10A and S10B, respectively. Bond has previously been shown to be required for both male and female fertility, and by *in situ* hybridization appears to be expressed during oogenesis in the main body follicle cells at stages 9 and 10 [42]. These genes may contribution in a manner that is not currently understood to mediate the production of lipid yolk, or they may function in the production of lipid-based signals that contribute to egg maturation.

### Pxt is required to synchronize egg maturation and eggshell production

Pxt mutations partially uncouple morphological development and gene expression. Yolk protein genes turn off normally in *pxt* mutant follicles, but vitelline membrane genes continue to be expressed longer than normal. Some chorion genes turn on earlier than normal, while the expression of others is delayed or prolonged. Many possible mechanisms may underlie these changes. However, we are particularly interested in the possibility that Pxt coordinates the production of PGs that interact with other mechanisms to precisely control egg maturation.

### Crosstalk between the germline and the soma

In all sexually reproducing organisms the growth and development of the somatic and germ cells are mutually dependent and must be coordinated. Such coordination requires bi-directional communication. Historically, somatic cells were thought to regulate follicle development, including maintaining meiotic arrest, promoting meiotic resumption, and suppressing oocyte transcription prior to nuclear maturation [43]. It has more recently been shown that the oocyte also signals to the soma [43], [44]. Oocyte signaling is necessary for follicular formation, and regulating the proliferation and differentiation of the somatic cells [21], [43], [45]. It is generally thought that the oocyte has a greater influence on the soma early in follicular development and this is reversed during the later stages [21].

There is emerging evidence that PG signaling coordinates germline and somatic development within mammalian follicles. While both oocyte and somatic maturation are delayed in COX2 knockout mice, it has been shown that the PGs are required in the soma for fertility [27]. Specifically, COX2 is required in the somatic cells for cumulus (somatic) cell expansion and survival. However, meiotic resumption is not controlled by PGs from the soma. These germline and somatic events must be coordinated for the follicle to be competent for fertilization. We have found that PG signaling is required for both germline and somatic development during Drosophila follicle development (this work and [25]). Fertility requires both of these signals. Specifically, PG signaling within the germline is necessary for mediating nurse cell dumping, the contractile process by which the oocyte is supplied with materials required for embryonic development, while PG signaling within the follicle cells is needed to regulate the timing of eggshell gene expression and subsequent eggshell structure. Thus PG signals, from insects to mammals, maintain the synchronized development of the germline and somatic cells within the individual follicle.

### Crosstalk between prostaglandins and steroid hormones

Female reproduction is regulated by a complement of hormones that are cyclically produced and secreted. One such hormone that interacts with PG signaling in mammals is oxytocin. Oxytocin plays critical roles in regulating the function of the corpus luteum, a transient endocrine organ that secretes hormones to regulate the menstrual cycle and the early stages of pregnancy. In the absence of pregnancy, PGF_2alpha_ stimulates the release of oxytocin to mediate luteolysis or the regression of the corpus luteum [46], [47]. During parturition, oxytocin and PGF_2alpha_ also play critical roles. Oxytocin initiates labor, inducing PGF_2alpha_, which maintains labor and dilates the cervix [46].

PGs and estrogen co-regulate each other in multiple cells types, including breast cancer cells. Breast tissue is the largest producer of estrogen in post-menopausal women; aromatase, Cyp19, leads to the production of estradiol. There is a high correlation between aromatase and COX2 expression in human breast cancer samples [48], [49]. Specifically, PGE_2_ signals via cAMP and PKA to stimulate a promoter upstream of *cyp19*, leading to increased aromatase expression [50]. Autocrine and paracrine feedback loops via estradiol subsequently increase PGE_2_ secretion [51], [52]. Therefore, in breast cancer cells, PG and estrogen signaling are intimately linked.

PGs and estrogen also interact in endometriotic tissue. Both PGE_2_ and PGF_2alpha_ are excessively produced in uterine and endometriotic tissues of women with endometriosis [53]. In the endometriotic stromal cells, PGE_2_ stimulates the expression of all the steroidogenic genes needed to synthesis estradiol from cholesterol. This occurs via PGE_2_ activation of cAMP/PKA signaling which upregulates of the expression of steroidogenic acute regulatory gene (*StAR*) and *cyp19* [54], [55], [56], [57]. The expression of these steroidogenic genes is regulated by Steroidogenic Factor 1 (SF1), a nuclear hormone receptor. PGE_2_ signaling leads to SF1 out competing other transcription factors, Chicken Ovalbumin Upstream Promoter Transcription Factor (COUP-TF) and Wilms' tumor-1 (WT-1), for binding to steroidogenic gene promoters [55]. Thus, PG signaling coordinates the expression of all steroidogenic genes.

Our results encourage future efforts to further establish the roles for PG signaling during Drosophila egg maturation and specifically, to learn how PGs are connected to steroid hormones. The Drosophila hormone ecdysone plays several critical roles during oogenesis. The loss of ecdysone signaling arrests follicle development at stage 8 [58], [59]. Additionally, ecdysone signaling is needed to control the onset of chorion gene amplification [58], and to activate eggshell gene expression via transcriptional regulation [19], [20]. Temporally programmed changes in ecdysone levels may contribute to the timed control of eggshell gene expression. The studies reported here provide a foundation for further dissecting the roles of Pxt and ecdysone-mediated signaling during late follicle development. If important aspects of these interactions have been conserved during evolution, the Drosophila ovary may emerge as a model for understanding the cellular and molecular changes underlying mammalian follicular maturation, endometriosis and infertility.

## Supporting Information

Figure S1 **Temporal control and tissue-specificity of follicle gene expression.** (A–B) The temporal expression patterns of 5 eggshell protein transcripts (A) and 5 non-structural transcripts (B) determined by RT-PCR. Their times of expression agree closely with the microarray data. (C–D) The tissue-specificity of the genes in A–B were assayed by RT-PCR using RNAs from the six indicated sources (1–6). Rps17 served as a loading control for all panels in C–D. In agreement with our RT-PCR tests, the FlyAtlas expression project (Chintapalli et al. 2007) found that 18 of 19 candidate eggshell genes were ovary-specific. Most of the temporally regulated non-structural genes were expressed more widely.
(TIFF)

Figure S2 **Effect of pxt mutation on the expression of selected non-egghell genes.** RT-PCR was used to assay transcript levels from the indicated genes during the stages 10B, 12 or 14 from wild type (*y w*) or pxt^f01000^ females (*pxt−/−*). Rps17 transcripts served as a loading control. pax = FBgn0041789; gstd1 = FBgn0001149; cg30382 = FBgn0050382; ste24a = FBgn0034176; cyp18a1 = FBgn0010383; cg30334 = FBgn0050334; yp3 = FBgn0004047; cyp4p2 = FBgn0033395; cg10013 = FBgn0038012; cg13937 = FBgn0035287; cg8027 = FBgn0033392; cyp4s3 = FBgn0030615; cg4066 = FBgn0038011, cg1681 = FBgn0030484.
(TIFF)

Table S1 **Expression of the well-characterized eggshell protein genes.** Table of transcript levels and stage-specifity as determined by microarray for the 30 well-characterized eggshell protein genes, and for three yolk protein genes. The previously characterized temporal expression (“Ref”) is listed under “Pattern.” References: ^1^Burke et al. (1987) Dev Biol 124: 441–450. ^2^Claycomb et al. (2004) Dev Cell 6: 145–155. ^3^Fakhouri et al. (2006) Dev Biol 293: 127–141. ^4^Parks et al. (1986) Dev Biol 117: 294–305. ^5^Parks and Spradling. (1987) Genes Dev 1: 497–509. ^6^Popodi et al. (1988) Dev Biol 127: 248–256. ^7^Yakoby et al. (2008) Dev Cell 15: 725–737.
(DOCX)

Table S2 **Putative new eggshell protein genes.** Table of transcript levels and stage-specifity as determined by microarray for the putative eggshell protein genes. ^1^Corresponds to Affy; gene model uncertain. ^2^See also Yakoby et al. 2008.
(DOCX)

Table S3 **Other genes regulated in late follicles by category.** Table of transcript levels and stage-specifity as determined by microarray for genes expressed temporally during late follicle development. ^1^See also Yakoby et al. 2008.
(DOCX)
