# Supplementary figures and images for: Drosophila Eggshell Production: Identification of New Genes and Coordination by Pxt
Source: PLoS One. 2011 May 26;6(5):e19943. doi: 10.1371/journal.pone.0019943 (PMC3102670; doi:10.1371/journal.pone.0019943)

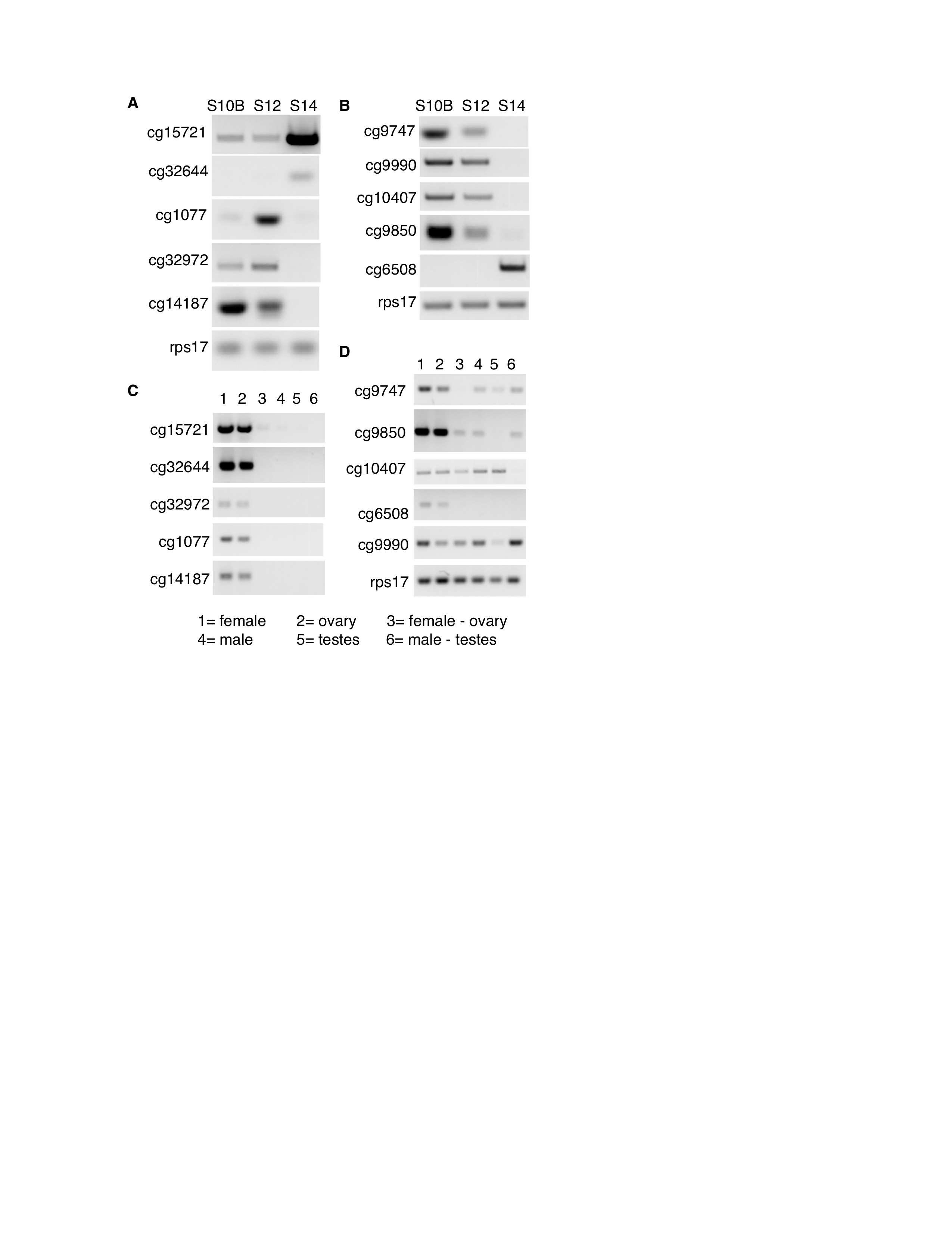

Supplement: Figure S1 — Temporal control and tissue-specificity of follicle gene expression. (A–B) The temporal expression patterns of 5 eggshell protein transcripts (A) and 5 non-structural transcripts (B) determined by RT-PCR. Their times of expression agree closely with the microarray data. (C–D) The tissue-specificity of the genes in A–B were assayed by RT-PCR using RNAs from the six indicated sources (1–6). Rps17 served as a loading control for all panels in C–D. In agreement with our RT-PCR tests, the FlyAtlas expression project (Chintapalli et al. 2007) found that 18 of 19 candidate eggshell genes were ovary-specific. Most of the temporally regulated non-structural genes were expressed more widely. (TIFF) [file pone.0019943.s001.tif]

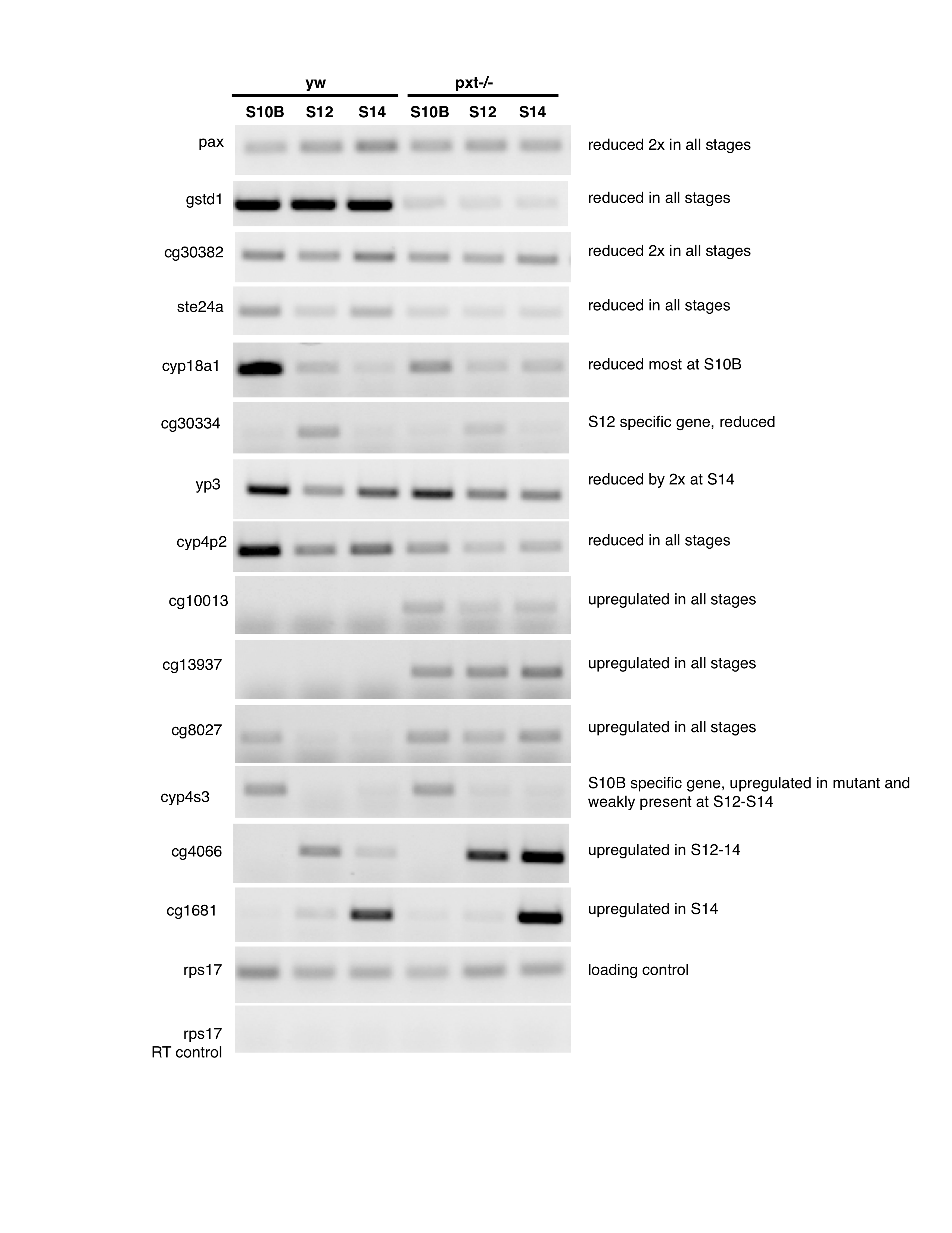

Supplement: Figure S2 — Effect of pxt mutation on the expression of selected non-egghell genes. RT-PCR was used to assay transcript levels from the indicated genes during the stages 10B, 12 or 14 from wild type (y w) or pxtf01000 females (pxt−/−). Rps17 transcripts served as a loading control. pax = FBgn0041789; gstd1 = FBgn0001149; cg30382 = FBgn0050382; ste24a = FBgn0034176; cyp18a1 = FBgn0010383; cg30334 = FBgn0050334; yp3 = FBgn0004047; cyp4p2 = FBgn0033395; cg10013 = FBgn0038012; cg13937 = FBgn0035287; cg8027 = FBgn0033392; cyp4s3 = FBgn0030615; cg4066 = FBgn0038011, cg1681 = FBgn0030484. (TIFF) [file pone.0019943.s002.tif]
