# Supplementary material for: Drosophila Eggshell Production: Identification of New Genes and Coordination by Pxt
Source: PLoS One. 2011 May 26;6(5):e19943. doi: 10.1371/journal.pone.0019943 (PMC3102670; doi:10.1371/journal.pone.0019943)
Supplement: Table S1 — Expression of the well-characterized eggshell protein genes. Table of transcript levels and stage-specifity as determined by microarray for the 30 well-characterized eggshell protein genes, and for three yolk protein genes. The previously characterized temporal expression (“Ref”) is listed under “Pattern.” References: 1Burke et al. (1987) Dev Biol 124: 441–450. 2Claycomb et al. (2004) Dev Cell 6: 145–155. 3Fakhouri et al. (2006) Dev Biol 293: 127–141. 4Parks et al. (1986) Dev Biol 117: 294–305. 5Parks and Spradling. (1987) Genes Dev 1: 497–509. 6Popodi et al. (1988) Dev Biol 127: 248–256. 7Yakoby et al. (2008) Dev Cell 15: 725–737. (DOCX) [file pone.0019943.s003.docx]

**Table S1: Expression of the well-characterized eggshell protein genes**

| Gene | Site | trans | S9-10A | S10B | S12 | S14 | Pattern | Ref |
| --- | --- | --- | --- | --- | --- | --- | --- | --- |
| Yp1 | 9B1 | RA | 16561.7 | 9820.7 | 224.3 | 460.9 | S9-10B |  |
| Yp2 | 9B1 | RA | 17789.6 | 7316.4 | 215.6 | 388.8 | S9-10B |  |
| Yp3 | 12B8 | RA | 16363.1 | 11819.0 | 358.5 | 910.8 | S9-10B |  |
| Vm34Ca | 34B7 | RA | 28007.4 | 27267.4 | 2517.5 | 1885.8 |  |  |
| Vm32E | 32E1 | RA | 5377.8 | 9002.3 | 56.9 | 70.8 |  |  |
| Vm26Aa | 26A9 | RA | 24388.8 | 25559.3 | 721.6 | 605.5 |  | 1 |
| Vm26Ab | 26A9 | RA | 22524.4 | 21207.0 | 621.5 | 525.5 |  | 1 |
| Fcp26Aa | 26A9 | RA | 4586.8 | 3073.0 | 60.1 | 63.5 |  | 1,3,6 |
| Fcp26Ac | 26A9 | RA | 2589.4 | 3135.0 | 77.2 | 37.2 |  | 1,3,6 |
| CG13992 | 26A | RA | 596.0 | 201.1 | 33.4 | 29.1 |  | 6 |
| CG31928^2^ | 22A2 | RA | 22.9 | 16.0 | 237.6 | 2632.4 |  | 3 |
| Cp36 | 7F1 | RA | 545.0 | 11613.8 | 27626.2 | 9991.4 |  | 4,5,7 |
| Cp38 | 7F1 | RA | 730.2 | 6353.3 | 25699.4 | 13256.3 |  | 4,5,7 |
| Cp7Fa | 7F1 | RA | 43.5 | 78.4 | 4792.6 | 925.4 |  | 5 |
| Cp7Fb | 7F1 | RA | 101.3 | 8170.4 | 5327.1 | 128.3 |  | 5,7 |
| Cp7Fc | 7F1 | RA | 107.1 | 8524.2 | 17365.2 | 204.4 |  | 5,7 |
| dec1 | 7C1 | RA | 12774.9 | 17855.9 | 3434.5 | 154.0 |  |  |
| yellow-g | 62D5 | RA | 60.2 | 342.8 | 16225.4 | 889.3 |  | 2 |
| yellow-g2 | 62D5 | RA | 121.3 | 585.6 | 18520.9 | 439.6 |  | 2,5 |
| CG13114 | 30B11 | RA | 114.5 | 185.2 | 17074.4 | 443.9 |  | 2 |
| CG11381 | 1F1 | RA | 51.9 | 1669.8 | 11390.8 | 651.7 |  | 3,7 |
| CG4009 | 89E10 | RA | 45.8 | 906.5 | 9935.3 | 408.2 |  | 3,7 |
| Fcp3C | 3C9 | RA | 187.9 | 8739.7 | 229.1 | 134.7 |  |  |
| CG15570 | 4B3 | RA | 31.8 | 61.0 | 5825.7 | 371.4 |  | 3 |
| Femcoat | 4B4 | RB | 39.6 | 12.6 | 1181.2 | 2608.0 |  |  |
| CG14796 | 2B2 | RA | 84.9 | 355.8 | 2379.2 | 482.0 |  | 3 |
| CG13083 | 37E4 | RA | 56.4 | 3.1 | 3302.3 | 5318.0 | S13-14 | 3 |
| CG13084 | 37E4 | RA | 39.6 | 5.3 | 2249.0 | 4223.0 | S13-14 | 3 |
| Cp18 | 66D14 | RA | 292.0 | 119.8 | 3158.9 | 37319.1 | S12-14 | 5 |
| Cp15 | 66D14 | RA | 113.6 | 356.3 | 461.3 | 29297.4 | S14 | 5,7 |
| Cp19 | 66D14 | RA | 820.8 | 4018.4 | 13186.6 | 27843.2 | S10B-14 | 5 |
| Cp16 | 66D14 | RA | 430.4 | 237.4 | 4218.9 | 31572.2 | S12-14 | 5 |
| CG12398 | 13A1 | RA | 912.9 | 41.6 | 10.7 | 17.9 | S9-10A | 3 |
