# Supplementary material for: Drosophila Eggshell Production: Identification of New Genes and Coordination by Pxt
Source: PLoS One. 2011 May 26;6(5):e19943. doi: 10.1371/journal.pone.0019943 (PMC3102670; doi:10.1371/journal.pone.0019943)
Supplement: Table S2 — Putative new eggshell protein genes. Table of transcript levels and stage-specifity as determined by microarray for the putative eggshell protein genes. 1Corresponds to Affy; gene model uncertain. 2See also Yakoby et al. 2008. (DOCX) [file pone.0019943.s004.docx]

**Table S2: Putative new eggshell protein genes**

| Gene | Site | Trans | S9-10A | S10B | S12 | S14 |
| --- | --- | --- | --- | --- | --- | --- |
| HDC17346^1^ | 4B4 |  | 294.8 | 284.5 | 7347.9 | 18066.4 |
| CG15571 | 4B4 | RA | 30.2 | 4.3 | 1110.5 | 425.8 |
| CG32774^2^ | 4B4 | RA | 10.8 | 21.2 | 5755.5 | 131.8 |
| CG15721 | 11D5 | RA | 21.4 | 42.1 | 45.2 | 1622.4 |
| CG32642 | 11D5 | RC | 12.4 | 3.2 | 102.3 | 2225.6 |
| CG32644 | 11D5 | RB | 8.2 | 11.1 | 90.2 | 434.8 |
| CG12716 | 11D5 | RA | 33.8 | 22.5 | 1365.8 | 237.2 |
| CG31926 | 22A2 | RA | 28.4 | 12.4 | 487.0 | 1758.1 |
| CG31661 | 22A2 | RA | 33.4 | 80.9 | 106.7 | 1109.3 |
| CG32602 | 12E7 | RA | 22.9 | 61.9 | 260.1 | 7658.9 |
| CG13113 | 30B11 | RA | 42.9 | 96.9 | 1742.3 | 276.8 |
| CG13299 | 65A6 | RA | 17.1 | 1025.6 | 7543.6 | 993.2 |
| CG1077 | 83D5 | RA | 2.4 | 82.9 | 3696.7 | 120.3 |
| CG32972 | 35A1 | RB | 2.9 | 133.3 | 1830.1 | 16.6 |
| CG14059 | 73E4 | RA | 2.1 | 8.3 | 5.5 | 645.8 |
| CG14187 | 76F2 | RA | 29.2 | 3171.3 | 262.5 | 29.3 |
| CG4066 | 87B9 | RA | 16.3 | 6.2 | 220.9 | 229.0 |
| CG12517 | 32C1 | RA | 1.9 | 13.6 | 16.2 | 338.0 |
| CG13998 | 26A8 | RA | 293.9 | 259.1 | 57.6 | 27.9 |
