# Supplementary material for: Drosophila Eggshell Production: Identification of New Genes and Coordination by Pxt
Source: PLoS One. 2011 May 26;6(5):e19943. doi: 10.1371/journal.pone.0019943 (PMC3102670; doi:10.1371/journal.pone.0019943)
Supplement: Table S3 — Other genes regulated in late follicles by category. Table of transcript levels and stage-specifity as determined by microarray for genes expressed temporally during late follicle development. 1See also Yakoby et al. 2008. (DOCX) [file pone.0019943.s005.docx]

**Table S3: Other genes regulated in late follicles by category**

| Gene | Site | trans | S9/10A | S10B | S12 | S14 |
| --- | --- | --- | --- | --- | --- | --- |
| lipid: |  |  |  |  |  |  |
| CG9747 | 99E1 | RA | 314.6 | 2673.2 | 735.4 | 42.4 |
| CG10163 | 65A10 | RA | 3.6 | 12.0 | 2066.1 | 65.5 |
| CG8303 | 53C9 | RA | 1672.6 | 1562.1 | 59.4 | 24.1 |
| CG14309 | 91A2 | RA | 1164 | 230.3 | 57.6 | 10.6 |
| CG2781 |  | RA | 81.7 | 1065.0 | 200.3 | 25.3 |
| Fatp | 31F4 | RA | 102.7 | 388.1 | 95.4 | 56.9 |
| CG31522^1^ | 82B | RB | 42.9 | 240.4 | 17.2 | 16.5 |
| Cyp4g1 | 1B3 | RA | 55.3 | 18.4 | 70.4 | 232.1 |
| CG7365 | 76F2 | RA | 6.1 | 189.7 | 31.4 | 17.9 |
|  |  |  |  |  |  |  |
| transporter: |  |  |  |  |  |  |
| CG7888 | 68A3 | RC | 11.5 | 55.2 | 991.8 | 69.2 |
| CG9990 | 98E1 | RA | 15.9 | 915.0 | 144.7 | 20.5 |
| CG9864 | 56F14 | RA | 37.9 | 165.4 | 477.0 | 746.1 |
| CG11147 | 26A1 | RA | 44.9 | 504.2 | 52.6 | 18.0 |
| CG5582 | 75A2 | RA | 281.4 | 89.7 | 9.0 | 24.0 |
| CG15890 | 12F4 | RA | 11.5 | 170.9 | 127.1 | 5.4 |
|  |  |  |  |  |  |  |
| cell cycle: |  |  |  |  |  |  |
| CG6921 | 94B6 | RB | 962.5 | 10.6 | 3.8 | 18.6 |
| CG5326 | 94B5 | RA,RB | 832.3 | 24.5 | 8.6 | 23.5 |
| CG4827 | 54B17 | RA | 473.2 | 279.7 | 34.6 | 14.6 |
| GstD2 | 87B8 | RA | 264.6 | 16.7 | 18.8 | 3.8 |
| CG4786 | 77C7 | RA | 233.2 | 7.5 | 20.9 | 23.8 |
| dally^1^ | 66E1 | RA | 149.8 | 51.8 | 9.9 | 1.7 |
| ana^1^ | 45A1 | RA | 27.2 | 14.8 | 3.5 | 11.5 |
|  |  |  |  |  |  |  |
| signals: |  |  |  |  |  |  |
| CG10407 | 89B | RA | 359.05 | 7711.7 | 2150.2 | 95.0 |
| ndl | 65B5 | RA | 2860.65 | 302.5 | 9.9 | 38.5 |
| Acer | 29D4 | RA | 241.55 | 405.6 | 1172.8 | 2388.7 |
| CG6704 | 50C20 | RA | 613.45 | 890.6 | 138.8 | 6.1 |
| tal |  |  | 503.6 | 650.9 | 114.1 | 17.3 |
| SP71 | 1A1 | RB | 315.6 | 183.7 | 36.4 | 17.0 |
| CG4250 | 58F4 | RA | 196.5 | 41.2 | 3.5 | 8.0 |
| sty | 63D2 | RB | 143.7 | 35.7 | 41.2 | 7.4 |

**Table S3 (cont.)**

| Gene | Site | trans | S9/10A | S10B | S12 | S14 |
| --- | --- | --- | --- | --- | --- | --- |
| enzymes: |  |  |  |  |  |  |
| amd | 37C1 | RB | 2.2 | 498.1 | 95.5 | 2.3 |
| CG9449 | 76B5 | RA | 87.85 | 370.2 | 61.5 | 26.3 |
| yellow-k | 71D4 | RA | 282.4 | 118.9 | 8.0 | 1.6 |
| fng | 78A1 | RA | 279.8 | 17.8 | 14.4 | 49.4 |
| Aph-4 | 100B1 | RA | 17.5 | 15.7 | 18.4 | 299.5 |
| CG17323 | 37B1 | RA | 228.9 | 31.8 | 17.9 | 56.6 |
| CG8776 | 49C1 | RD | 177.45 | 19.5 | 7.4 | 10.9 |
| CG9449 | 76B5 | RB | 42.1 | 151.8 | 23.7 | 9.2 |
| CG7565 | 66B5 | RA | 82.7 | 37.6 | 21.4 | 27.8 |
| Cyp18a1 | 17D1 | RB | 39.0 | 317.1 | 130.3 | 67.3 |
| Cyp305a1 | 76D3 | RA | 26.5 | 51.3 | 183.6 | 179.1 |
|  |  |  |  |  |  |  |
| proteases: |  |  |  |  |  |  |
| CG9850 | 59F7 | RA | 70.8 | 1727.7 | 424.6 | 36.2 |
| CG6508 | 32B1 | RA | 2.5 | 2.8 | 3.5 | 910.4 |
| CG5367 | 31D10 | RA | 281.3 | 182.5 | 31.3 | 25.8 |
|  |  |  |  |  |  |  |
| inhibitors: |  |  |  |  |  |  |
| Cys |  | RA | 55.2 | 53.5 | 788.5 | 359.7 |
| Timp | 86A1 | RA | 156.8 | 31.0 | 170.3 | 23.5 |
| CG15418 | 24A2 | RA | 9.0 | 66.1 | 784.2 | 47.3 |
|  |  |  |  |  |  |  |
| adhesion: |  |  |  |  |  |  |
| ImpL2^1^ | 64B2 | RA | 1581.5 | 456.5 | 246.1 | 132.1 |
| Cad74A^1^ | 74A | RB | 27.35 | 1075.9 | 1700.4 | 99.5 |
| PH4EFB | 99F7 | RA | 1110.75 | 172.4 | 29.4 | 17.6 |
| CG3624 |  | RA | 648.15 | 329.8 | 45.1 | 22.7 |
| wbl | 56C4 | RA | 547.05 | 54.4 | 2.9 | 4.6 |
| PH4alphaPV | 100A1 | RA | 27.85 | 97.9 | 465.2 | 71.7 |
| LanA | 65A8 | RA | 273 | 42.4 | 21.7 | 23.9 |
| RH62830 |  |  | 304.7 | 66.1 | 39.0 | 21.5 |
| CG31869 | 32A5 | RA | 306.85 | 18.3 | 9.8 | 6.3 |
| Cad99C^1^ | 99C | RA | 261.3 | 155.6 | 179.8 | 22.0 |
| LanB2 | 67C2 | RA | 188.3 | 50.2 | 39.2 | 17.6 |
| BM-40-SPARC | 97D3 | RA | 155.05 | 36.8 | 24.1 | 50.6 |
| pip^1^ | 76A6 | RA | 152.45 | 17.0 | 11.9 | 6.0 |
| LanB1 | 28D1 | RA | 116.7 | 44.6 | 12.3 | 14.7 |

**Table S3 (cont.)**

| Gene | Site | trans | S8-9 | S10B | S12 | S14 |
| --- | --- | --- | --- | --- | --- | --- |
| transcription: |  |  |  |  |  |  |
| srp^1^ | 89B3 | RA | 26.55 | 104.2 | 1198.7 | 103.4 |
| Eip75B | 75B | RB | 629.65 | 267.4 | 262.7 | 337.4 |
| HmgZ | 57F9 | RB | 380.3 | 111.6 | 50.1 | 46.1 |
| ttk | 100E1 | RF | 209.4 | 386.0 | 52.8 | 33.9 |
| Eip74EF | 74EF | RA | 32.35 | 381.2 | 136.9 | 20.9 |
| tj | 37E3 | RA | 306.65 | 73.4 | 10.4 | 26.3 |
| CG2052 | 102B6 | RB | 78.1 | 211.0 | 26.3 | 38.5 |
| jim | 80A3 | RB | 188.05 | 207.4 | 22.9 | 7.4 |
| Egfr | 57F1 | RA | 140.75 | 118.0 | 25.5 | 30.6 |
| CrebA | 71D | RA | 97.6 | 79.5 | 0.5 | 4.2 |
|  |  |  |  |  |  |  |
| neural: |  |  |  |  |  |  |
| mfas | 87A7 | RB | 1487.75 | 391.6 | 15.4 | 13.5 |
| nemy | 49C1 | RD | 52.45 | 47.4 | 313.5 | 642.9 |
| CG1698 | 46B3 | RA | 10 | 27.0 | 20.9 | 506.5 |
| cpo | 90D1 | RA | 116.95 | 289.8 | 44.7 | 11.9 |
| Oamb | 92F2 | RB | 5.3 | 24.8 | 17.6 | 273.0 |
| bnb | 17E | RB | 11.2 | 8.6 | 229.0 | 25.4 |
| comm2 | 71F1 | RA | 52.9 | 203.4 | 13.0 | 10.9 |
| CG15324^1^ | 7C1 | RA | 109.95 | 122.0 | 20.2 | 3.2 |
| Sur | 31B1 | RA | 13.5 | 5.9 | 157.7 | 19.9 |
| Eaat1 | 30B1 | RB | 2.1 | 4.1 | 77.1 | 6.1 |
|  |  |  |  |  |  |  |
| unclassified: |  |  |  |  |  |  |
| Ov | 65F3 | RA | 143.9 | 5628.4 | 7523.0 | 136.4 |
| Transposon.34 | |  | 2497.1 | 234.8 | 39.3 | 30.5 |
| CG13737 | 70C5 | RA | 28.8 | 63.7 | 544.0 | 58.9 |
| CG14059 | 73E4 | RA | 2.1 | 8.3 | 5.5 | 645.8 |
| CG6579 | 33D2 | RA | 11.3 | 16.0 | 9.6 | 624.2 |
| CG9336 | 38F1 | RA | 30.7 | 45.5 | 39.8 | 382.7 |
| CG11347 | 64B6 | RD | 4.1 | 1.3 | 17.3 | 226.3 |
| CG8913 | 89E7 | RA | 27.4 | 39.5 | 79.8 | 233.8 |
| Msr-110 | 64D3 | RA | 11.05 | 553.0 | 477.9 | 9.6 |
| CG13117 | 30C | RA | 64.45 | 103.9 | 421.9 | 199.1 |
| gk | 75B11 | RB | 62.15 | 399.9 | 76.7 | 68.8 |
| Iris | 21F1 | RA | 241.1 | 36.7 | 35.2 | 55.1 |
| CG9322 | 87F13 | RA | 110.35 | 14.6 | 23.0 | 10.3 |
| GM03661 | 9F |  | 187.15 | 39.7 | 24.7 | 74.0 |
